# The Current Concept and Evidence-Based Practice in the Base of the First Metacarpal Bone Fracture

**DOI:** 10.7759/cureus.51600

**Published:** 2024-01-03

**Authors:** Saran Malisorn

**Affiliations:** 1 Orthopaedics, Naresuan University, Phitsanulok, THA

**Keywords:** trapeziometacarpal joint injury, fracture base of the first metacarpal bone, first metacarpal bone fracture, rolando fracture, bennett fracture

## Abstract

Repetitive instances of sudden injuries to the first metacarpal bone can affect thumb movement. These injuries typically occur after vertical impact to the thumb. The treatment for these injuries should focus on restoring the structure and biomechanics of the trapeziometacarpal joint, with surgery being recommended for optimal results. Bennett's fracture involves the bifurcation of the bone into two distinct fragments, characterized by a smaller fragment and a larger counterpart. Rolando fracture is associated with a fracture of the base of the first metacarpal bone, typically divided into three parts. An extra-articular fracture involves the metacarpal bone of the thumb. Conservative treatment outcomes have been found to be unsatisfactory when the fracture is displaced. Therefore, surgery methods such as minimally invasive surgery, open reduction, and arthroscopic surgery have been proven to be effective. Surgical techniques for bone procedures include pin fixation, direct screw fixation, indirect screw fixation, and mini-plate fixation. Additionally, the prognosis of this condition depends on the ability to restore joint mobility during rehabilitation.

## Introduction and background

This article aims to comprehensively explore injuries to the base of the first metacarpal bone, focusing on their impact, treatment strategies, and outcomes in the context of thumb functionality.

Injuries to the base of the first metacarpal bone, often resulting from forceful impacts or trauma to the thumb, are prevalent in current medical conditions. Common causes include falls onto the outstretched hand or direct blows to the base of the metacarpal bone. These injuries can significantly impact thumb flexibility, highlighting the importance of effective management. Unlike thumb lesions, the approach to treating acute injuries to the metacarpal bone in the thumb varies based on their proximity to the trapeziometacarpal joint. This variability arises due to the compensatory effects of adjacent joints. Extra-articular fractures at the base of the first metacarpal bone can tolerate minor abnormalities in rotation or slight deviations in the frontal and sagittal planes [1]. The depression or collapse of the first base metacarpal bone, resulting from a shortened and 30° varus angulation, can be compensated for by the excessive extension of the metacarpophalangeal joint [2]. However, it is crucial to recognize that incomplete dislocations and fractures of the joint can lead to short-term impairment, emphasizing the need for a nuanced and targeted treatment approach. This study seeks to explore the multifaceted aspects of these injuries, considering their impact, treatment strategies, and outcomes on thumb functionality.

Biomechanics

**Figure 1:**
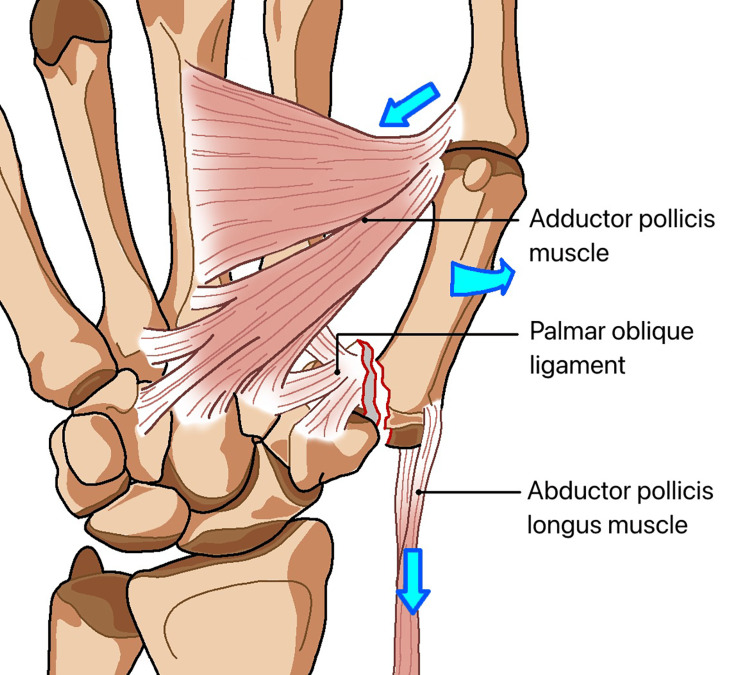
Bennett’s fracture refers to the movement and fracture of the base of the first finger joint. The abnormality is caused by the adductor muscle on the inner side, exerting an oblique downward and rotational force on the first metacarpal bone. The first metacarpal bone is brought close to the remaining components of the forearm and is held in place by the tendon of the hand and the abductor pollicis longus muscle.

The mechanism of injury involves compression along the axis of the thumb metacarpal bone, which can lead to partial bending and fractures of the other bones within the joint. The compression is caused by the traction of the abductor pollicis longus (APL) muscle, adductor pollicis muscle, and volar beak ligament, as shown in Figure 1. The volar ulnar corner of the metacarpal base remains stable due to the presence of a strong volar oblique ligament.

Wu et al. found that during a key pinch, the carpometacarpal (CMC) joint can withstand forces up to 12 times greater than the force applied [3]. The combination of daily high forces and increased movement in the trapeziometacarpal joint can lead to increased pressure on the joint, which may contribute to the development of arthritis in the CMC joint. This highlights the potential significance of surgical intervention in cases of Bennett's fracture.

Injuries frequently occur at the base of the first metacarpal when pressure is applied along its axis while flexing the thumb after an injury. This type of injury is estimated to occur in approximately 4% of wrist and hand fractures [4]. All treatments, including endoscopic techniques and percutaneous approaches, carry a risk of dislocation and potential damage to blood vessels and nerves [5].

The optimal treatment for metacarpal fractures of the thumb is still a subject of debate, as recent studies have shown unfavorable outcomes with only reduction and closed casting. Until 1980, conservative treatment was reported to yield satisfactory outcomes for these fractures [6-8]. Untreated sudden injuries to the metacarpal bone of the thumb can lead to instability and carry a significant risk of complications. Various osteosynthesis techniques such as wire fixation, headless screw fixation, locking plates, and screws, have been described for the reduction of the first base metacarpal bone [9,10].

The branch of the median nerve on the palmar surface of the skin should be protected during the procedure to avoid injury. When approaching the posterior aspect, there is a risk of damaging the branch responsible for sensory perception of the radial nerve. The main risk of plate fixation is damage to the radial artery. Closed reduction with percutaneous K-wire fixation through the skin can often lead to pin tract infections [11], which may result from the loss of fracture reduction. This can occur more frequently when the K-wire is less stable through the skin compared to fixation.

**Figure 2:**
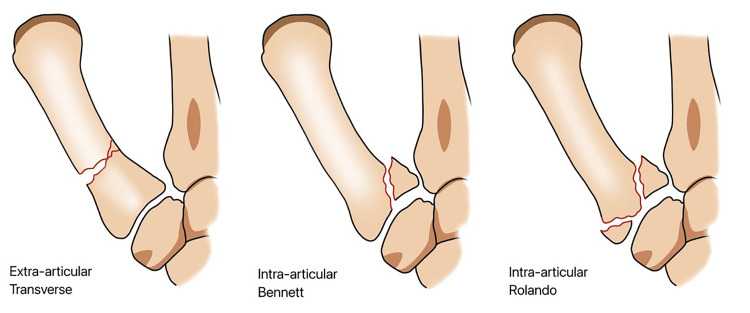
Extra-articular fracture, intra-articular fracture (Bennett's fracture), and Rolando's fracture.

Fractures can be categorized into two types: extra-articular fractures (Figure 2) and articular fractures, such as Bennett's fracture (which can be small or large) and Rolando's fracture (which is a three-part fracture). The varus and subluxation of the thumb's posterior compartment are caused by the actions of the adductor pollicis and APL [12,13]. While true instability is not a normal condition, it can contribute to a diminished sense of stability.

Radiography

**Figure 3:**
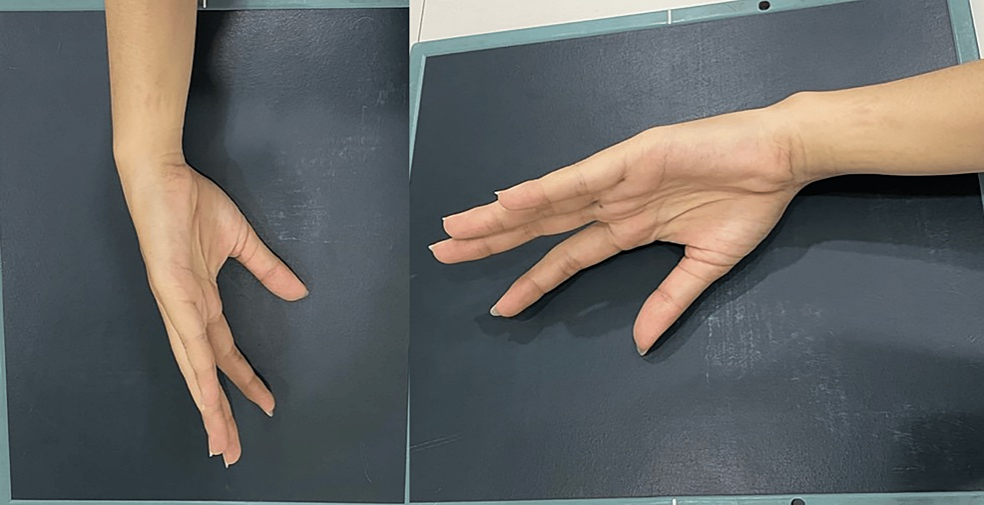
True lateral view of the thumb obtained by pronating the hand 20° and then angling the beam 15° to 20° distal.

**Figure 4:**
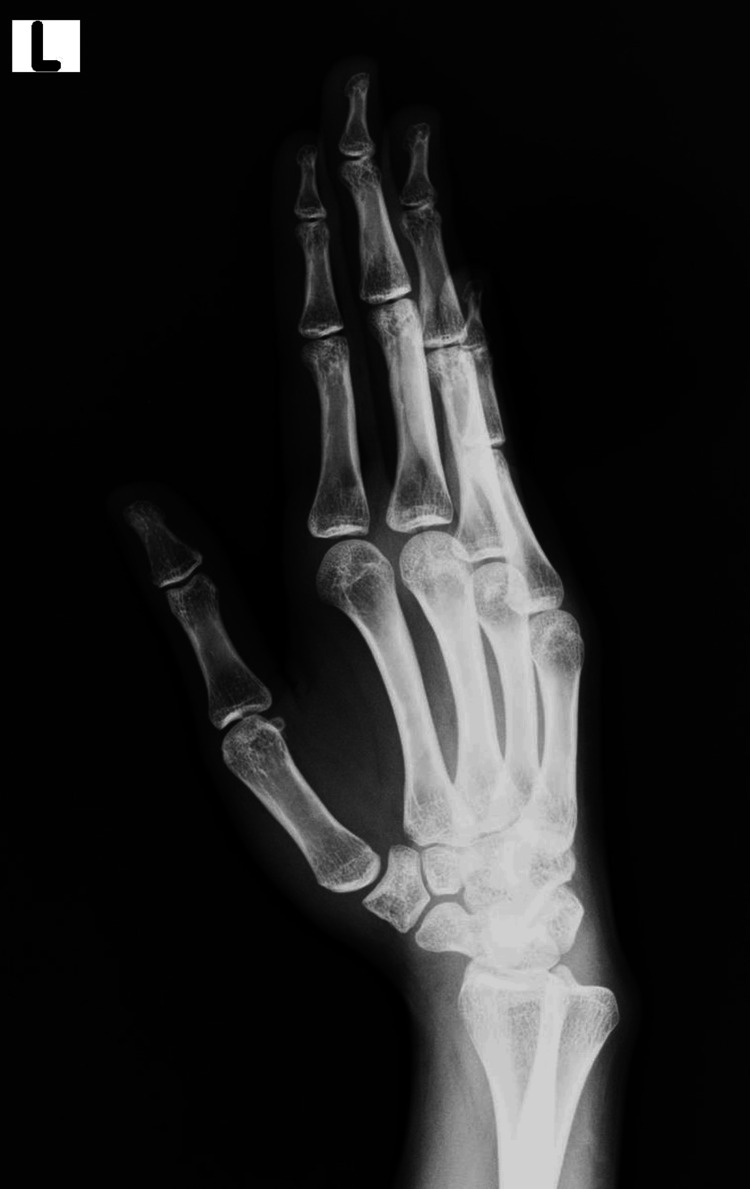
Radiographic results of the true lateral view of the thumb obtained by pronating the hand 20° and then angling the beam 15° to 20° distal.

Radiographic imaging is necessary for diagnosis, and every patient should receive posteroanterior, lateral thumb, and oblique views as described by Billing and Gedda. This is essential for accurately assessing the joint base of the first metacarpal bone in the first CMC joint [14]. To achieve a true lateral perspective, the hand should be extended outward at 20°, followed by angling the light beam at 15° to 20° (Figures 3, 4). X-ray images can also be used to assess the outcome of ligamentotaxis in reducing the force of compression [15]. Additionally, computed tomography (CT) scanning can be utilized to evaluate comminuted fractures.

## Review

Bennett’s fracture

Definition

Fracture and movement of the metacarpal bone in the thumb have distinct characteristics in terms of severity and abundance in articles discussing causes and treatment, similar to the movement of pure trapeziometacarpal. Edward H. Bennett first documented this phenomenon in 1882, where fractures of various sizes resulted in the separation of both bones. The anterior base angle of the metacarpal bone in the thumb, which is the smallest component, would be locked in place (Figure 2) and connected to the trapeziometacarpal joint by the oblique extensor line. The metacarpal bone of the thumb undergoes two movements and is the most important component of the metacarpal APL group, which results in the first occurrence of subluxation of the dorso-radial trapeziometacarpal joint. Subsequently, adduction will narrow down the first locking plate under the influence of the central thenar muscles. Bennett's fracture can be classified into two types, based on the size of the anterior fragment: major fracture and minor fracture.

The Assessment of Patients

During assessments, male patients are predominantly observed to exhibit symptoms of Bennett's fracture. It is worth noting that not all fractures necessarily present with symptoms [16,17]. The diagnostic process relies on a thorough history and physical examination. Patients often directly report the hand that has been impacted or their ability to bear weight along the axis. They immediately report pain and swelling at the base of the first metacarpal bone. Upon examination, tenderness at the base of the CMC joint and crepitus, indicating attempted movement, can be observed. Additionally, a reduced range of motion may become apparent. The evident abnormality of the shaft of the first metacarpal bone at the lower part may result from the movement of the metacarpal bone.

Classifications

**Figure 5:**
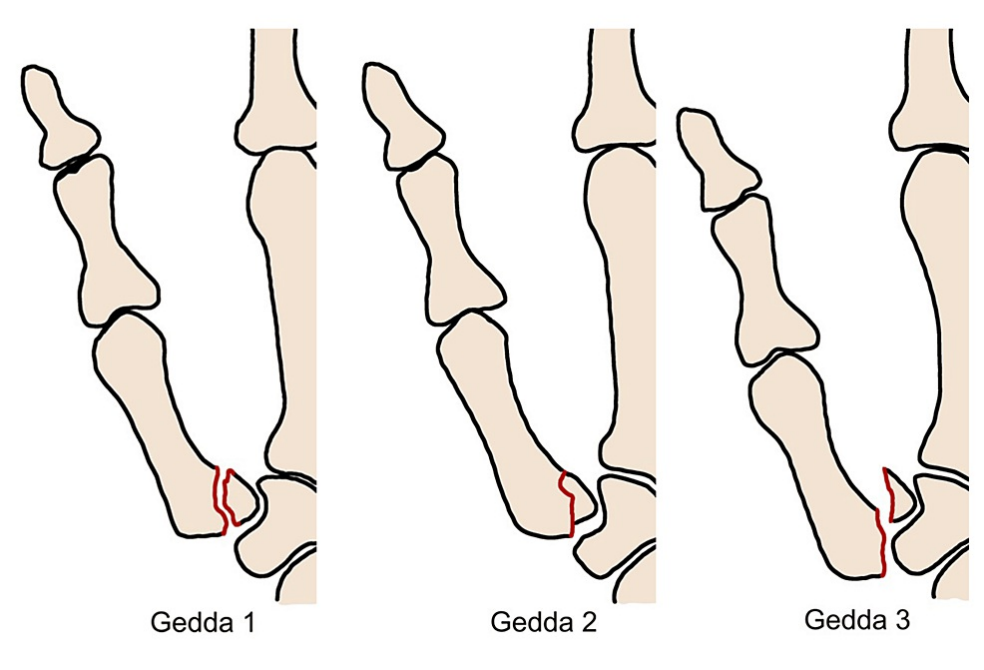
Type 1: A fracture with a large single ulnar fragment. Type 2: An impaction fracture without subluxation of the thumb metacarpal. Type 3: A small ulnar avulsion fracture fragment in association with metacarpal dislocation.

In 1952, Gedda published a series on the non-operative management of Bennett's fracture and proposed a classification system consisting of three types [18]. Type 1 is characterized by a single large fracture line and a subfragment at the base of the metacarpal bone. Type 2 is a fracture that occurs from a direct impact or injury without any separation of the metacarpal bone. Type 3, ulnar avulsion fracture, is a small-sized fracture that is associated with the movement of the metacarpal bone, as shown in Figure 5 [18].

Treatment

The initial step in treating a fracture of the first metacarpal bone of the thumb is reduction, which involves correcting the initial plate contraction and releasing the pollicis longus abductor to extend the thumb. Direct pressure is applied to the thumb metacarpal within the anatomical snuffbox while using a fluoroscope to achieve reduction and prevent joint incongruity. The reduction of this joint fracture should be done as accurately as possible. The second strategy is to ensure that the reduction process continues smoothly [19]. There is a lack of consensus on the optimal treatment approach, with some advocating for conservative treatment based on motions and others recommending open reduction and internal fixation (ORIF) using a closed reduction technique. The primary goal of treatment is to achieve a high-quality joint reduction, although the relationship between arthritis and functional outcomes is still debated [19].

Non-surgical Treatment

**Figure 6:**
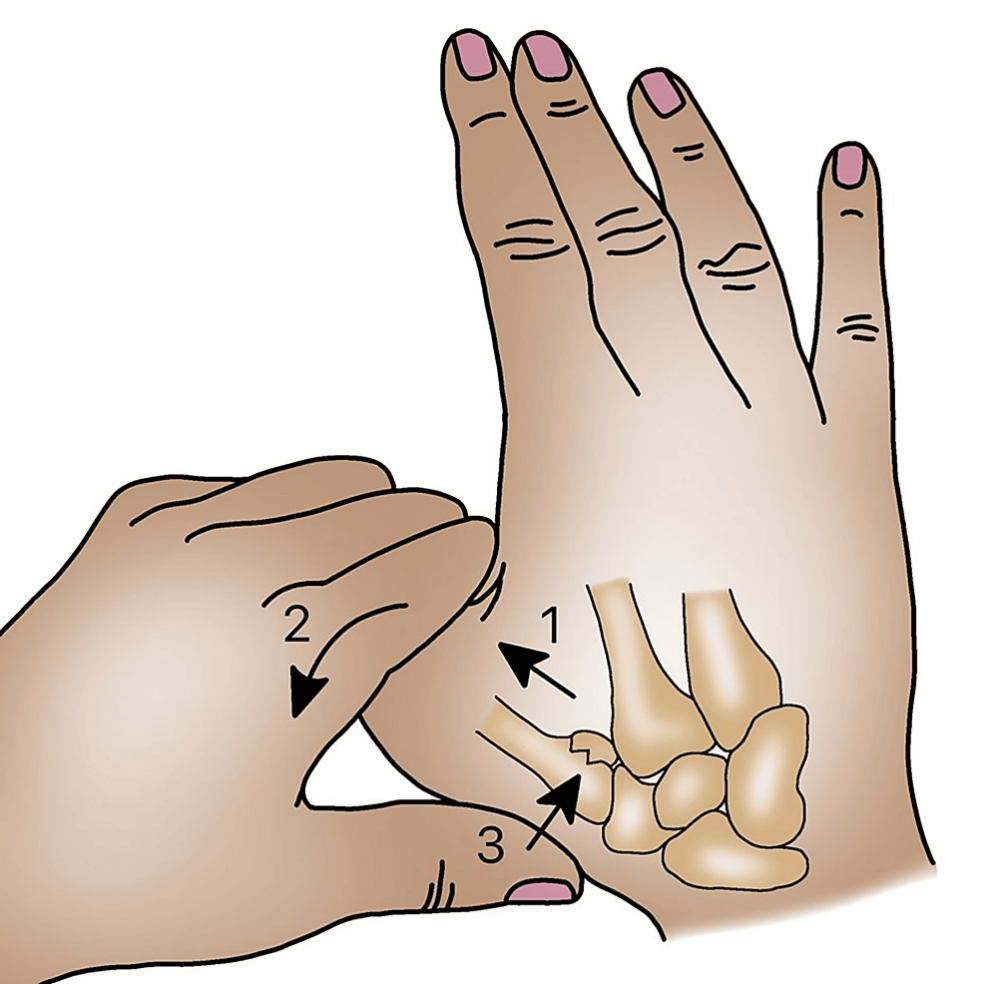
Reduction technique in Bennett’s fracture.

In the past, closed reduction and casting were the primary treatment methods for these injuries, typically lasting for four to six weeks [20-22]. The reduction can be achieved by applying axial traction, extending the metacarpal bone, and applying radial pressure above the base of the metacarpal bone (Figure 6). After reduction, a splint can be used to maintain the position, with pins securing the thumb splint. In the 1960s, long-term studies on non-operative management revealed frequent loss of reduction and subsequent post-traumatic arthritis. This led surgeons to shift toward surgical fixation [20,21]. Gedda and Moberg initially reported successful non-operative treatment in 49 out of 54 patients [17]. However, they eventually became proponents of surgical fixation early on due to evidence of decreased loss and radiographic findings of arthritis in 41 patients [17]. While radiographic evidence and clinical findings may show loss of motion and signs of arthritis, some datasets still show high patient satisfaction levels [20]. In the study by Thurston et al. in 1993, 76 cases of Bennett's fracture were followed up for seven years. In cases where a good reduction was not achieved, the results were generally satisfactory, with only one patient requiring subsequent fusion of the first CMC joint. However, all patients experienced loss of finger flexion and extension, as well as decreased grip strength. The authors concluded that surgical treatment should be performed, reserving non-operative treatment for non-displaced Bennett's fractures. It is recommended to achieve a reduction with less than 1 mm displacement in cases of Bennett's fracture [23].

Surgical Treatment

**Figure 7:**
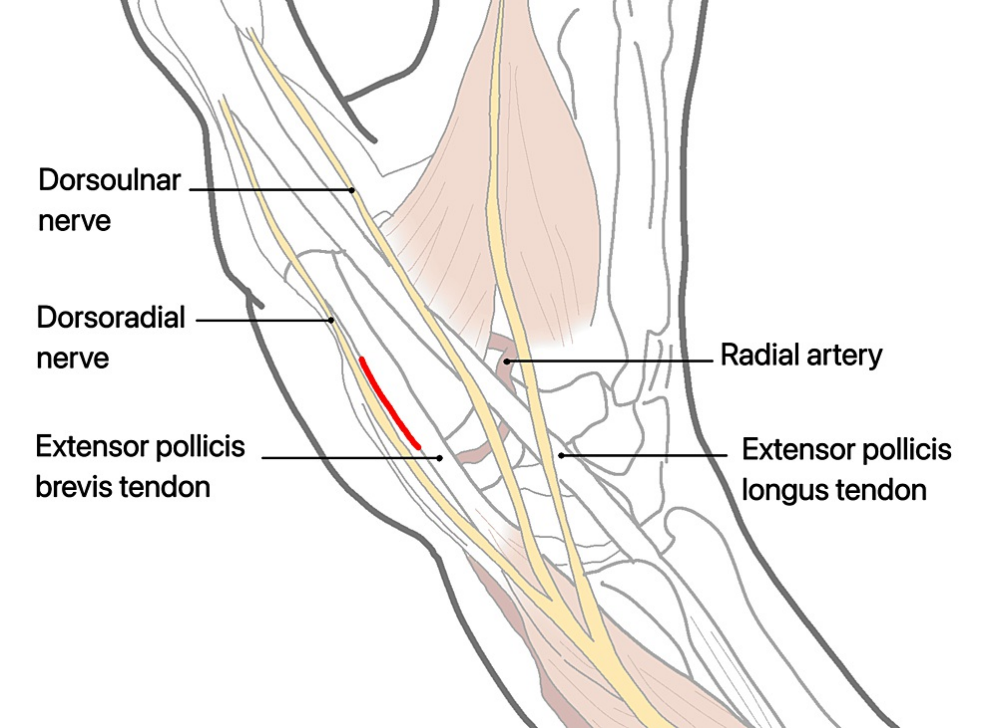
Make a 1 cm long dorsal skin incision over the base of the thumb before insertion of the K-wire.

**Figure 8:**
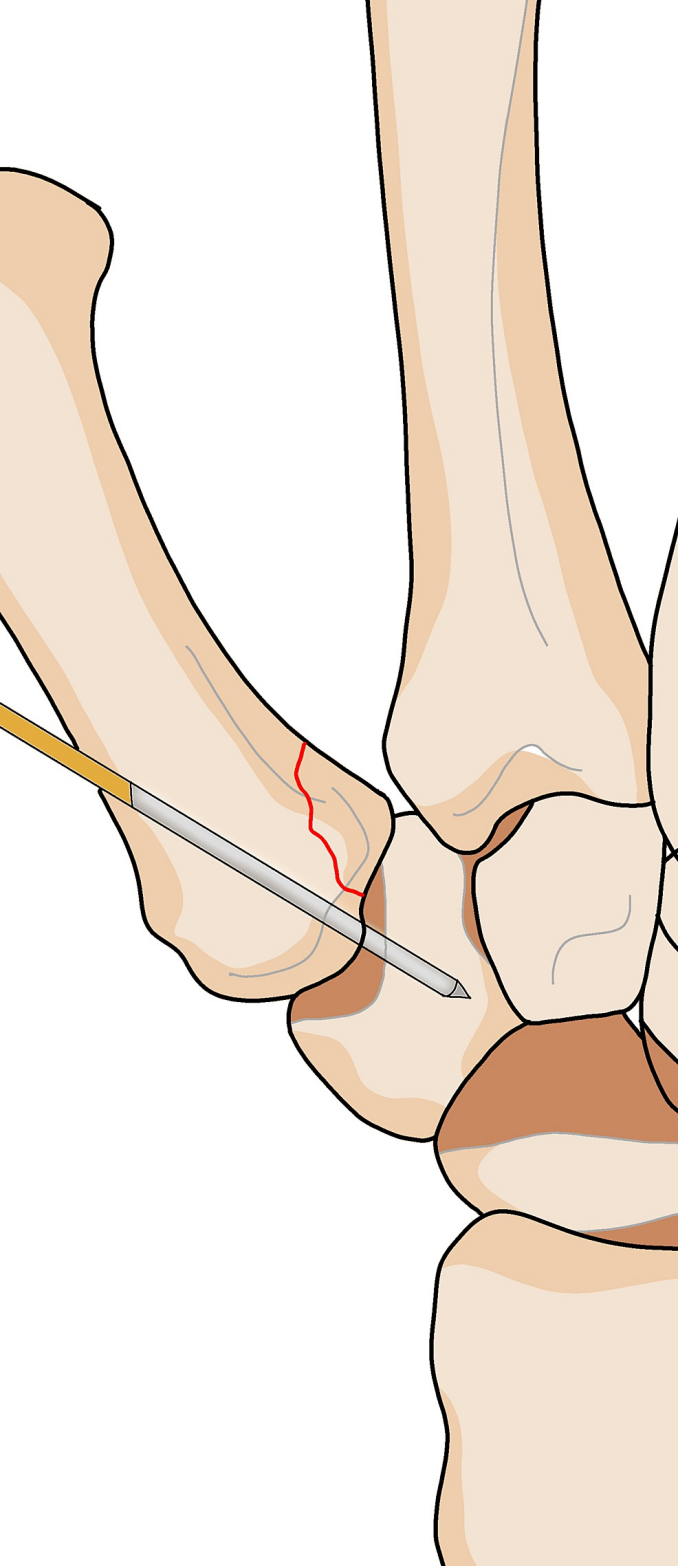
The first K-wire through predetermined entry points on the base of the first metacarpal and the trapezium.

**Figure 9:**
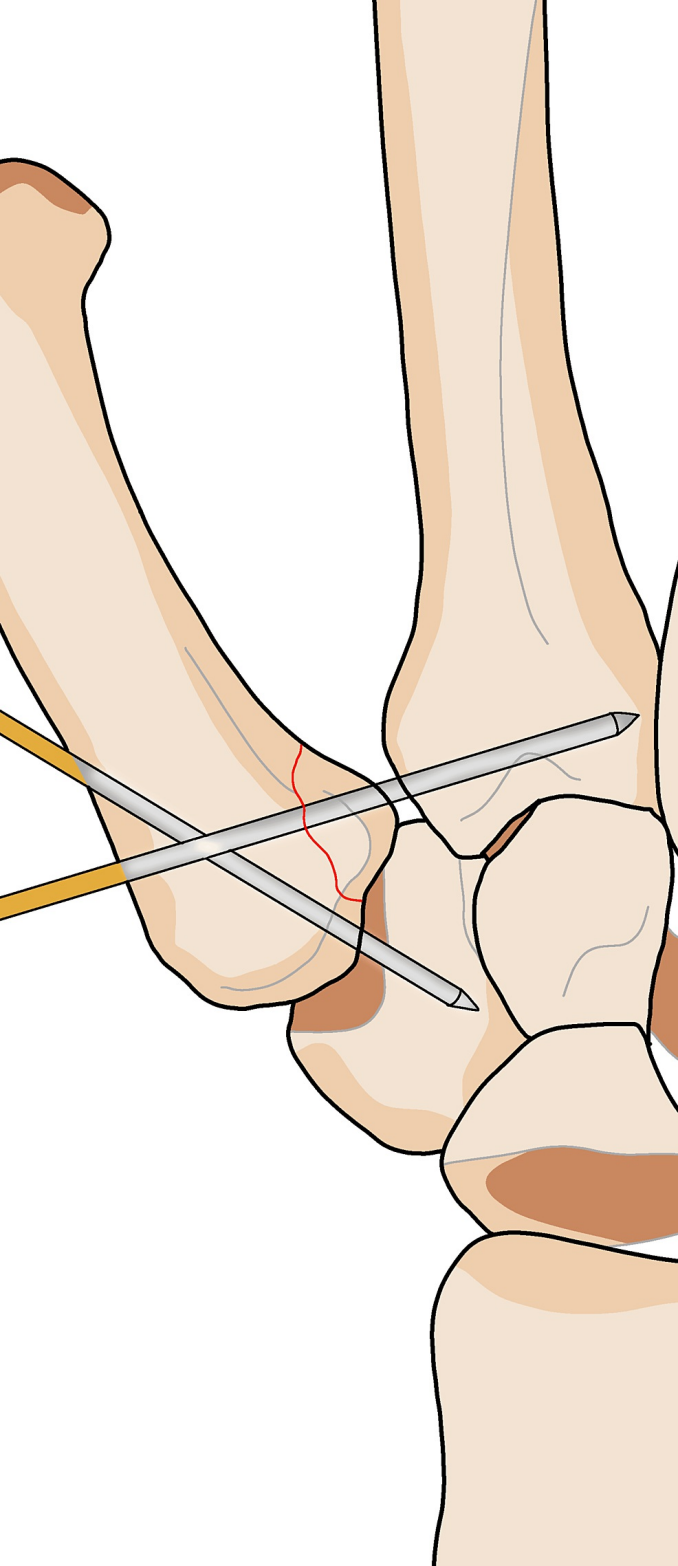
The second K-wire transfix into the fracture by passing the K-wire through the fragment.

Osteosynthesis pins fixation, screws [12,24,25], or plates is a technique described for use during open reduction, arthroscopy, or Bennett's fracture fixation. Pins are used for direct [24,26] or indirect [25] fixation, either crossing or non-crossing the fracture site in most of the percutaneous osteosynthesis procedures [27-30]. The base of the first metacarpal bone had fixation using a Kirschner wire (K-wire) combined with an external fixator [31]. The Iselin technique, which involves the fixation of the joint between bones and articulations [32], is one of the most effective treatment methods. However, it can lead to complications such as secondary displacement, arthritis, post-traumatic arthritis, infection of the skin and deep tissues, and limitations in finger flexion. Skin drilling pins are commonly used due to their ability to reduce the risk of muscle-tendon adhesion [32,33]. All techniques of osteosynthesis pin fixation through the skin carry a risk of infection or nerve damage [34], and all techniques that avoid the trapeziometacarpal joint are at risk of joint stiffness, infectious arthritis, or post-traumatic arthritis [24,35]. To avoid these issues, the of use screws instead of pins has been proposed. Prepare the surgical site using sterile techniques, including draping and cleaning the area, and suggest making a 1 cm long dorsal skin incision over the base of the thumb before inserting the K-wire. This step is commonly performed in surgical procedures involving K-wire insertion to provide adequate access (Figure 7). Gently dissect through the subcutaneous tissue to expose the underlying structures, providing a clear view of the base of the thumb. Proceed with the insertion of the K-wire through the designated entry point, guided by fluoroscopy, and transfix the base of the first metacarpal to the trapezium using K-wires. Insert the wires through the bone fragments to stabilize the fracture. Perform closed reduction techniques to manipulate and realign the fractured fragments. Carefully insert the first K-wire through predetermined entry points on the base of the first metacarpal and the trapezium, ensuring proper alignment. This may involve fluoroscopic guidance (Figure 8). Carefully insert the second K-wire transfix into the fracture by passing the K-wire through the fragment, stabilizing it in the desired position. Use fluoroscopy or other imaging methods to confirm the reduction and appropriate fixation. Close the incision with appropriate sutures or adhesive strips, ensuring proper wound closure (Figure 9).

ORIFs have the advantage of facilitating ligament tugging or capsular insertion. Anatomically reduced joint fractures under direct vision and eventual open osteosynthesis. The posterior approach (Cantero technique) and the palmar approach (Gedda-Moberg technique) are two possible surgical techniques for ORIF. Pins can be used for steps performed through the skin, but opening up the fractured area allows for the use of more durable techniques such as plates [30,31] and screws, which can be either conventional [12,31] or cannulated compression screws [36].

The use of internal fixation is favored over arthroscopy due to its minimally invasive approach, which reduces the risk surrounding soft tissue and blood vessels, minimizing tendon adhesion and bone necrosis [37]. Internal fixation allows for direct control of joint surface reduction, which is inversely related to the development of post-traumatic arthritis, and aids in the capsular insertion or separation of tendons. The main drawback of internal fixation with a single K-wire is the instability and lack of complete halt to secondary motion. Some believe that the inability to achieve reduction through closed fixation is a limitation of percutaneous screw fixation [34]. Pin fixation without surgical incisions near the fractured bone can be used to reduce the size [35]. Dual percutaneous screw fixations can replace the single K-wire to enhance stabilization during the procedure [37]. Tension contributing to tendon formation reduces joint mobility, while compressing the fracture position leads to a diminished gap between the fracture components. The reduced joint occurs by using a pin or screw insertion beneath the articular cartilage to prevent component displacement and achieve compression. Internal fixation and reduction using arthroscopy for Bennett fractures have disadvantages such as increased costs, issues related to the learning curve, and longer installation and removal times. The disadvantages of internal fixation and reduction using arthroscopy outweigh the significant benefits, as internal fixation improves joint reduction quality and reduction increases traction along the axis of the thumb used for arthroscopic setup.

Indication

The involvement of the articular surface and the presence of an articular step-off component are important factors in determining the need for surgery. ORIFs are commonly used to treat significant Bennett and Rolando fractures [38]. Leclère et al. found long-term success in treating 21 large Bennett fractures using open screw osteosynthesis with an articular step-off >1 mm. However, this approach increases the risk of post-traumatic arthritis, and one patient experienced subluxation for the second time within nine weeks after surgery [39]. Lutz et al. reported 32 cases of large-sized Bennett's fractures treated with open screw fixation and joint stabilization through percutaneous drilling. After an average duration of seven years, the overall strength of the hand reached up to 89% compared to the contralateral side. The type of treatment did not significantly impact the final clinical outcomes or the severity of inflammation of the blood vessels after injury, although the minimally invasive surgery group may have a higher incidence of abnormalities in the thumb metacarpal bone [40]. After surgery, it is typically necessary to use a removable splint for approximately four weeks to allow immediate mobilization if stable fixation is achieved.

The comparative study of ORIF and close reduction percutaneous pin fixation (CRPP) has been conducted by several researchers. In 1994, Timmenga et al. compared 18 patients (seven patients with CRPP and 11 patients with ORIF) with a mean follow-up of 10.7 years [41]. They found no correlation between treatment methods and the progression of arthritis. However, there was a significant correlation between the level of reduction and the changing progression of arthritis. Out of the seven patients treated with CRPP, five who showed accurate reduction still experienced ongoing progression of arthritis [41]. In the 1990s, Kjaer-Petersen et al. achieved excellent results with reduction (<1 mm displacement) in their study of 31 patients with an average follow-up time of 7.3 years [42]. They found a correlation between excellent reduction without symptoms and radiographic imaging results. Out of the 18 patients, 15 showed excellent reduction without symptoms compared to six out of 13 patients with residual motion, and there was a correlation with the radiographic imaging results. The progression of arthritis supports the need for "clear reduction if necessary" through open reduction. No significant differences were found in clinical outcomes or arthritis resulting from radiographic imaging during treatment. The occurrence of a fixed adduction deformity was significantly higher in cases treated with open reduction, while CRPP was preferred for displacement less than 1 mm. ORIF was reserved for fractures that could not be adequately aligned or when Kirschner wires could not be inserted into the fractured metacarpal bone of the thumb [43].

Recently, there has been an explanation of the reduction in fracture size of arthroscopy and internal fixation of Bennett fractures [44,45]. The theoretical advantages of ORIF include reduced damage to surrounding soft tissues and blood vessels, as well as direct visualization of the joint surface for better alignment. Yin et al. reported on seven patients who underwent arthroscopic treatment, concluding that this technique helps in reducing joint stepping but does not guarantee the stability of fixation [45].

**Figure 10:**
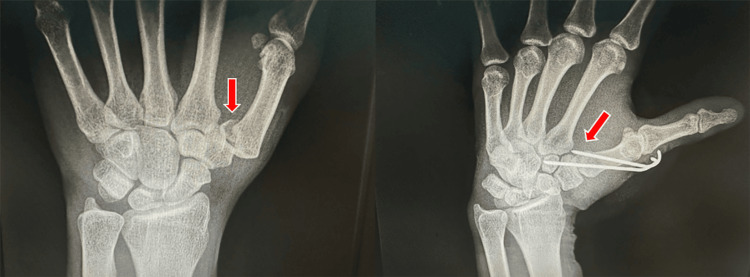
Percutaneous fixation for Bennett's fracture through the trapeziometacarpal joint.

**Figure 11:**
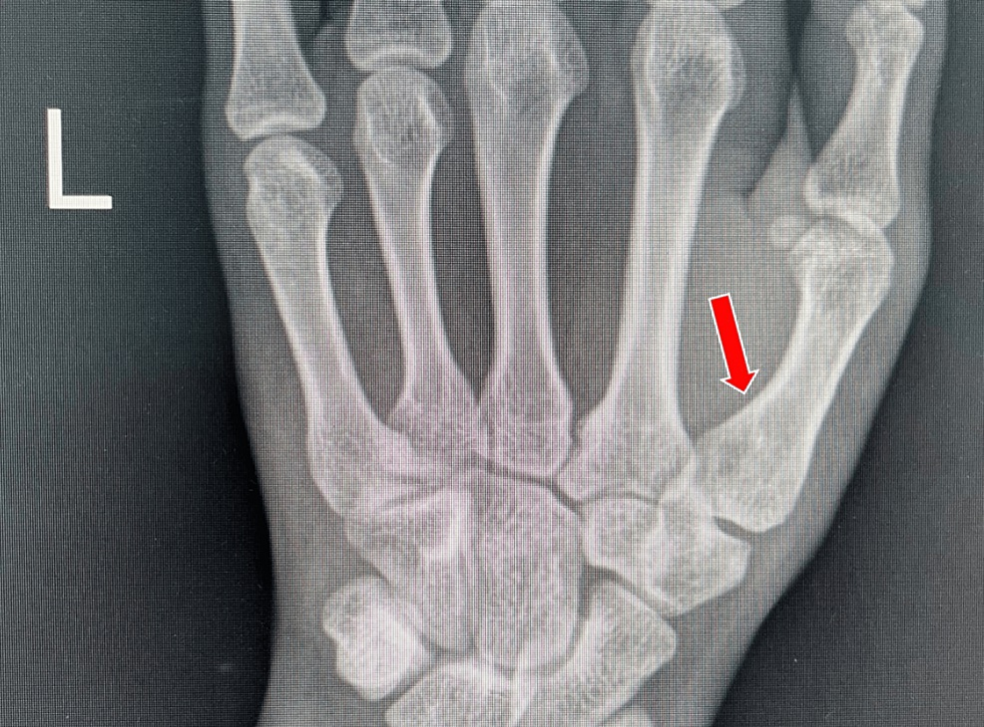
Anteroposterior view after removing the K-wire.

**Figure 12:**
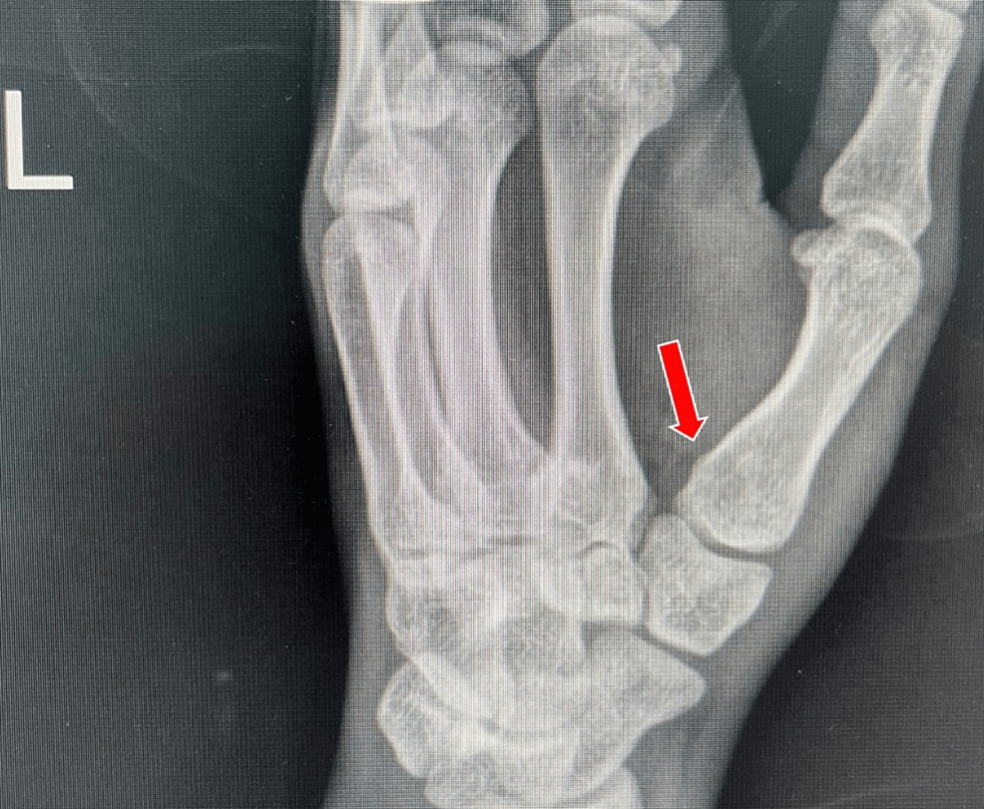
Oblique view after removing the K-wire.

Authors recommend reducing the fracture displacement through external manipulation and percutaneous fixation if the anterior fragment is too small to be directly addressed. Various positions for wires have been suggested, including through the trapeziometacarpal joint (Figures 10-12) [46,47] or in an extra-articular location, across the space between fingers. Inserting pins on the inner side of the joint may result in increased damage to the articular surface, despite achieving opposition of the thumb in all patients and the average strength is 80% compared to the contralateral side. Several authors have reported their outcomes, on average, 18 months after percutaneous joint fixation. The Iselin technique may lead to complications, such as in the latest follow-up, 16 out of 21 patients with trapeziometacarpal joint involvement experienced a reduction. For example, the index finger extension may be irritated if the tip of the pin protrudes from the dorsal aspect of the gap between the index and middle fingers. Greeven et al. reported on an average follow-up of 24 months, and one patient out of 25 patients who underwent surgery for the fracture at the base of the metacarpal bone experienced infection at the pin site three times, and had visual abnormalities [48].

Prognosis

The prognosis for Bennett's fracture treated in the acute phase is generally good, especially when there is a small fracture fragment [49]. However, the distance on the articular surface is longer than 1-2 mm, there is a larger fragment involved, and the prognosis is less favorable [50]. In displacement of the Bennett metacarpal shaft CMC articular step-off of more than 1 mm and articular surface involvement <20%, recommend CRPP. If the anterior marginal fragment is large enough for internal fixation >20% of the articular surface, recommend ORIF.

Rolando fracture

Definition

Rolando fracture, a three-part, intra-articular fracture above the base of the metacarpal bone of the thumb, was first described by Italian surgeon Silvio Rolando in 1910 [51]. It is characterized as a "T" or "Y" shape [52]. The direction, quantity, and movement of the tendon fracture distinguish Rolando's fracture from Bennett's fracture. The diaphysis and epiphysis are sometimes distributed throughout most areas. The epiphysis is divided into two parts, with the second line of fracture occurring within the vertical joint (Figure 2). The fracture involves three or more parts above the base of the metacarpal bone of the thumb and is often comminuted [53]. Central coupling is frequently infiltrated. Each component of the fracture has its own specific experience displacement. The first line is closed due to the central thenar muscle pulling out a large distal diaphysis in an adduction movement by spreading the thumb. The lateral portion of the epiphyseal fragment is elevated and removed, while the posterior oblique part retains the central portion of the epiphyseal fragment together with the trapeziometacarpal joint. Injuries often occur due to compressive forces along the axis of the metacarpal bone while the trapeziometacarpal joint is in flexion [54]. These fractures often lead to subluxation or instability of the trapeziometacarpal joint due to the tension force of the APL tendon [55]. Surgery is necessary to address the internal instability [56].

Treatment

The primary goal of treating a Rolando fracture is to achieve anatomical reduction, ensuring that the fractured bones align with their original anatomical position. This is followed by stable fixation for early mobilization and appropriate range of motion of the trapeziometacarpal joint, with the aim of reducing pain [57,58]. However, treating a Rolando fracture can be challenging due to the inherent nature of instability and comminuted patterns. There is also the risk of complications such as loss of reduction, joint incongruity, or the development of osteoarthritis [54,59].

**Figure 13:**
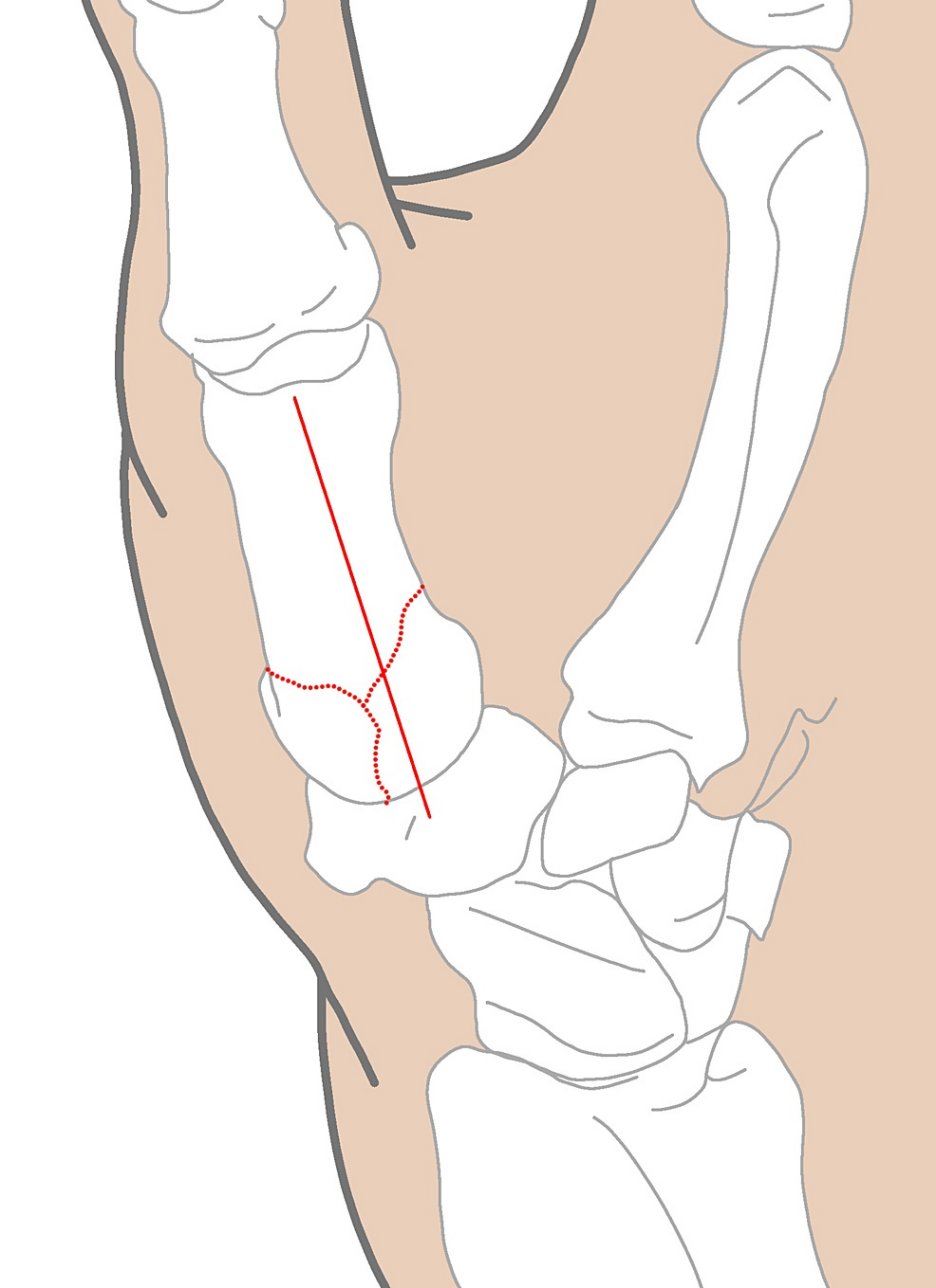
For Y- or T-shaped patterns in the frontal plane, a straight dorsal approach is preferred.

**Figure 14:**
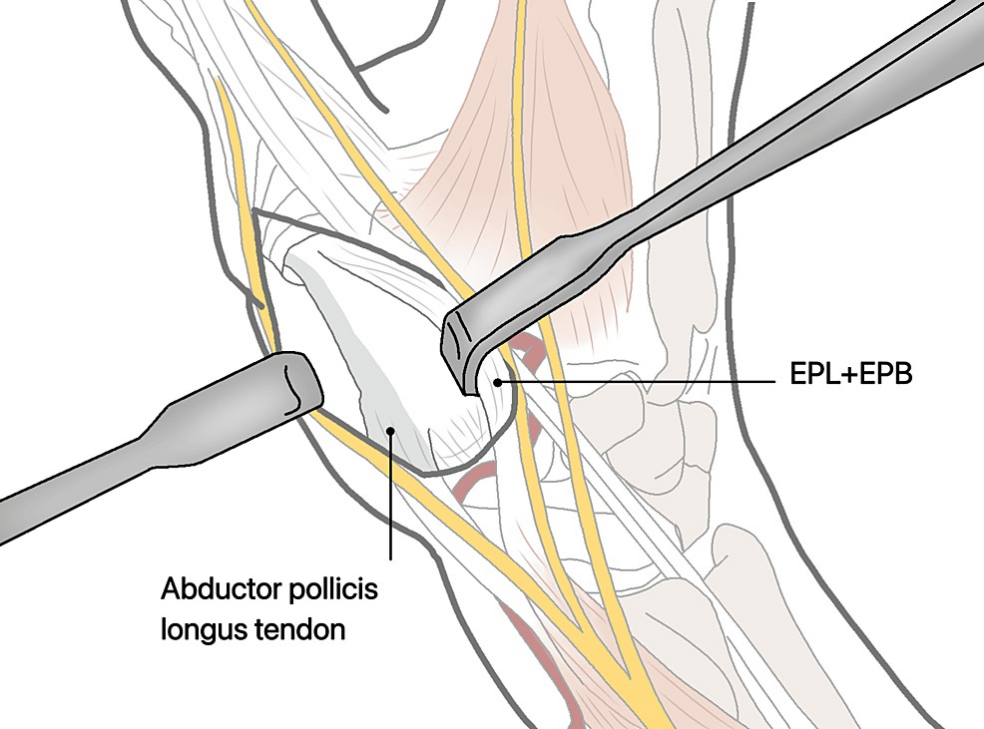
In the radial side of the EPB, incise the fascia and retract both tendons in an ulnar direction. EPL: extensor pollicis longus; EPB: extensor pollicis brevis.

**Figure 15:**
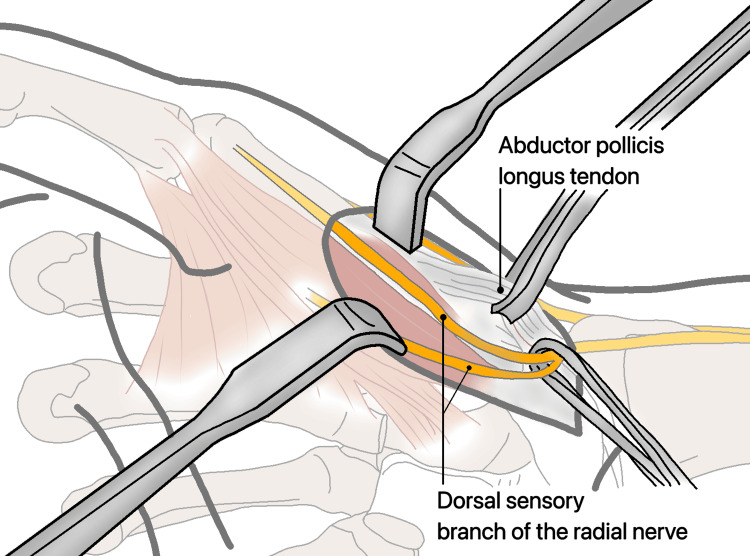
The division of the dorsal sensory branch of the radial nerve and the APL tendon. APL: abductor pollicis longus.

**Figure 16:**
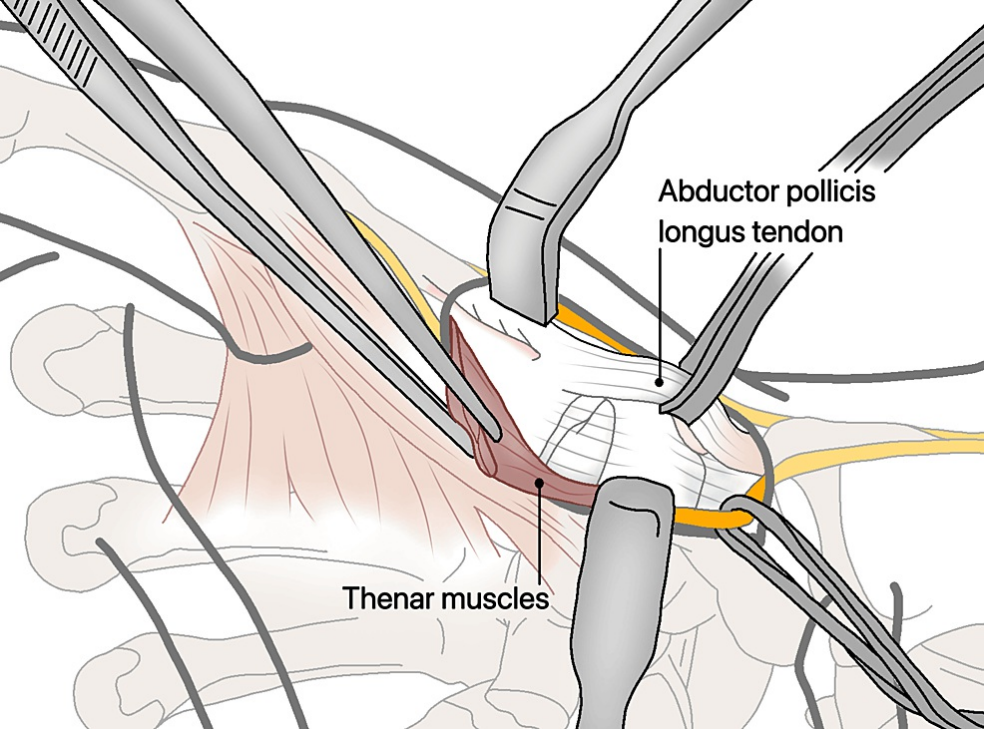
Detached from their origins at the base of the first metacarpal and reflected in a palmar direction.

**Figure 17:**
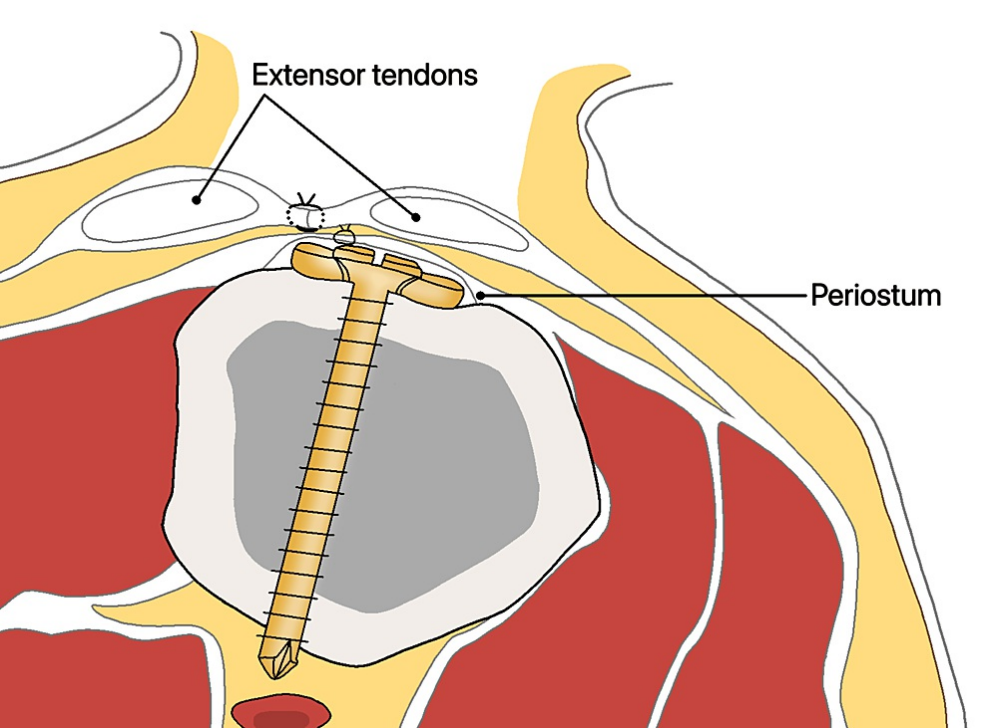
Lifting the periosteum and covering the implant with it as far as possible helps to minimize contact between the extensor tendons and the implant.

**Figure 18:**
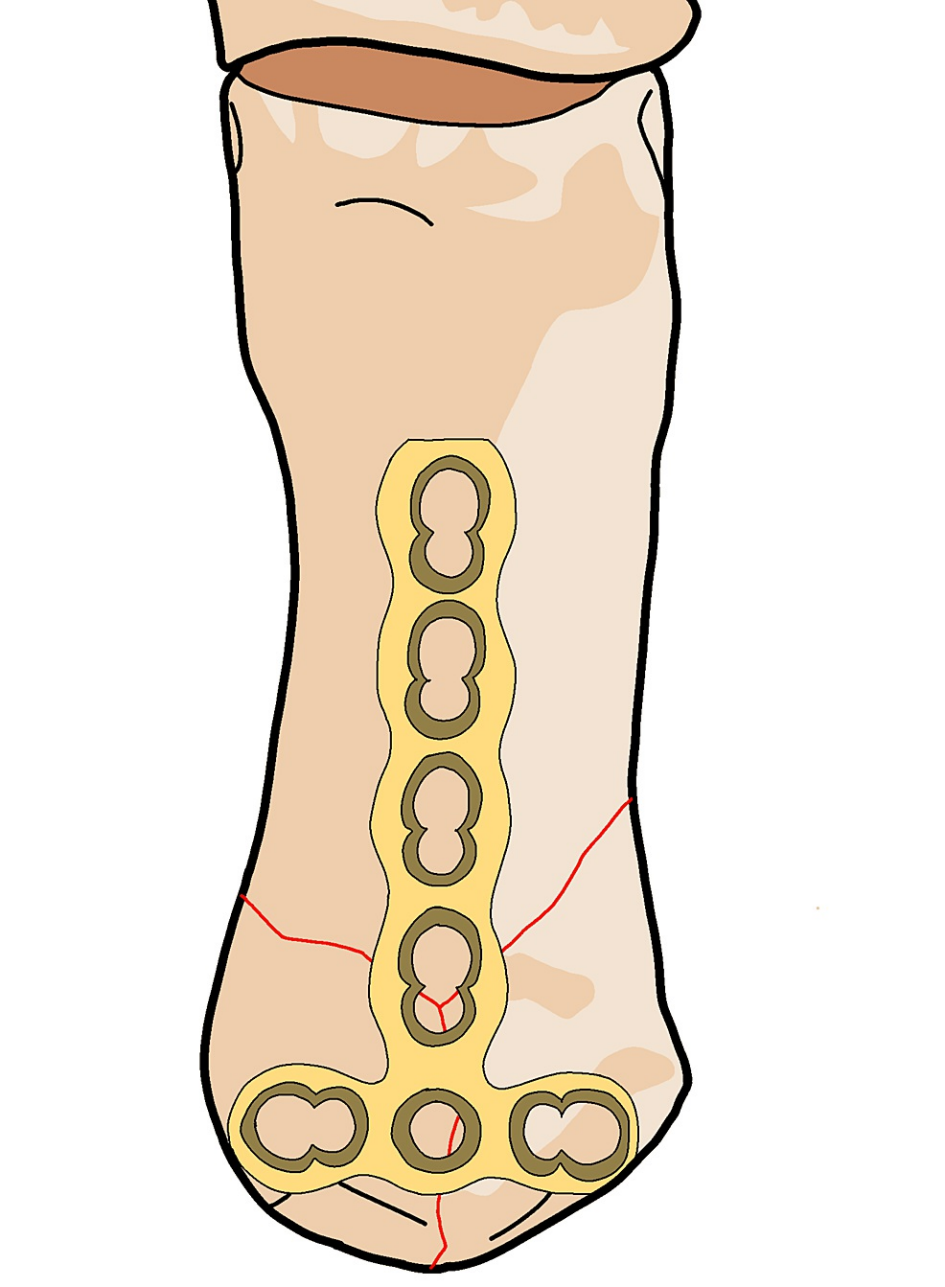
A 2.0 mm locking compression plate (LCP) T-shaped is best suited for these fractures.

**Figure 19:**
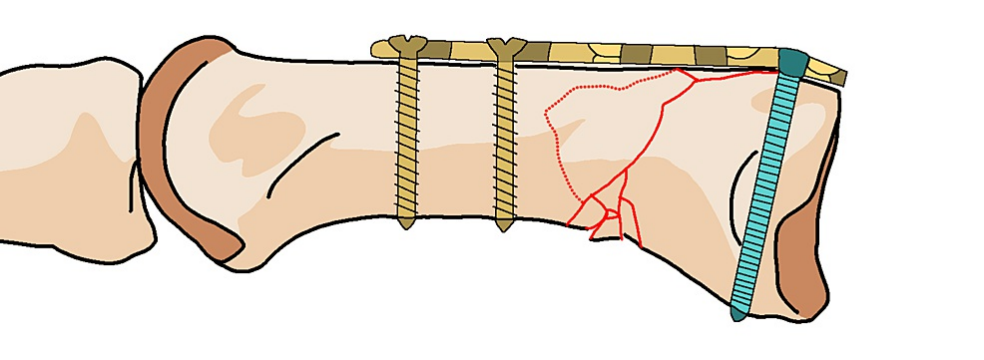
Locking compression plate to length so that two screws can be fixed in the distal diaphyseal fragment.

Achieving a precise reduction is necessary in three-part fractures to prevent joint incongruity, which can lead to arthritis. However, external treatment methods often cannot achieve reduced fractures, and conservative treatment and percutaneous osteosynthesis often yield unsatisfactory results. Fixation of the trapeziometacarpal joint may not provide an acceptable reduction and can lead to joint degeneration due to iatrogenic causes, as mentioned by some authors [60]. A straight dorsal incision based on the location and extent of the Y- or T-shaped Rolando fracture. This incision should provide optimal exposure for the planned fixation. Make a straight dorsal incision over the Rolando fracture site, aligning it with the planned approach (Figure 13). Dissect through the subcutaneous tissue and muscle layers to expose the fractured bone segments, taking care to handle the unique anatomy of the thumb (Figure 14). Manipulate and reduce the fractured fragments into the desired anatomical alignment, considering the comminuted nature of a Rolando fracture (Figure 15). Choose an appropriate fixation device, a plate suitable for the Y- or T-shaped Rolando fracture. Insert and secure the plate using screws in a manner that stabilizes the unique fracture pattern (Figure 16). Carefully position the pre-bent locking compression plate (LCP) over the fracture site, aligning it with the bone's anatomy. Identify the optimal locations for screw insertion in the distal diaphyseal fragment. Based on the measured length of the fragment and the planned screw positions, adjust the length of the 2.0 mm LCP accordingly (Figure 17). Insert screws through the pre-drilled holes in the LCP into the distal diaphyseal fragment. Use screws of appropriate length to achieve secure fixation. Verify the reduction and fixation using fluoroscopy or other imaging methods (Figure 18). Complete the surgical procedure by closing the incisions and ensuring proper wound closure. Provide postoperative care instructions, including details on follow-up appointments, rehabilitation, and activity restrictions (Figure 19).

**Figure 20:**
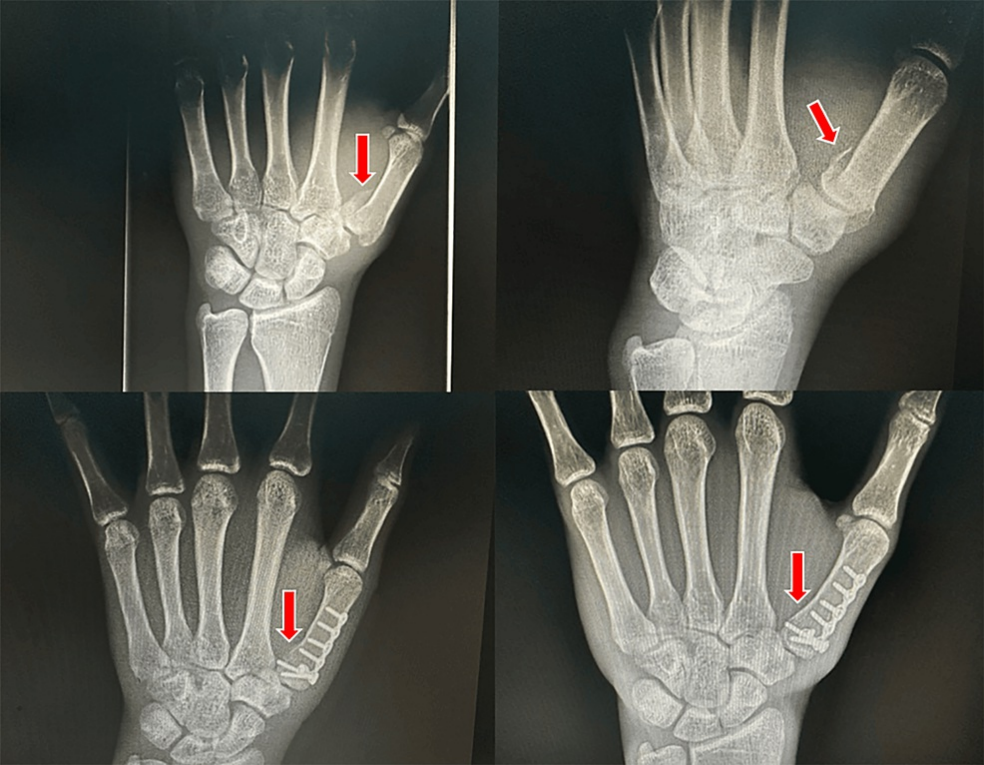
Locking plate with screws for Rolando fracture.

Regardless of the method used, it is recommended to follow certain steps to restore regularity and stability of the joint, whether through open surgery or minimally invasive arthroscopic procedures. The fracture is reduced according to anatomical principles, and the diaphysis and epiphysis are stabilized with small pins or locking plates (Figure 20).

After surgery, it is recommended to use removable splints immediately if the structure is stable enough. These splints should be worn for four weeks [61]. Tsai et al. achieved satisfactory radiological reduction without complications in five patients who underwent surgery for Rolando fracture. After a three-month follow-up, patients who achieved bone unions showed nearly complete restoration of opposition, abduction, pinch, and grip strength. The use of a hooked embracing plate is considered a good and safe option for surgical fixation in patients with Rolando fractures compared to traditional methods such as lag screw or mini-plate fixation. It provides rigid fixation, as confirmed by fine radiological assessment, and facilitates early mobilization for better functional outcomes [62]. Uludag et al. reported on seven patients with Rolando fractures who underwent ORIF with a mini-plate or screw. All patients achieved fine bone union, anatomical reduction, and early mobilization. They also had full trapezio-metacarpal joint movement and an acceptable loss of grip and pinch strength of less than 20% [63]. Mumtaz et al. reported on nine patients with Rolando fractures who underwent ORIF with a mini-plate. The functional outcomes were good except for one patient who experienced pain and poor range of motion and had stage 3 Eaton-Littler osteoarthritis. Four patients had their implants removed due to tenderness [64].

The extra-articular fracture of the base of the first metacarpal bone

**Figure 21:**
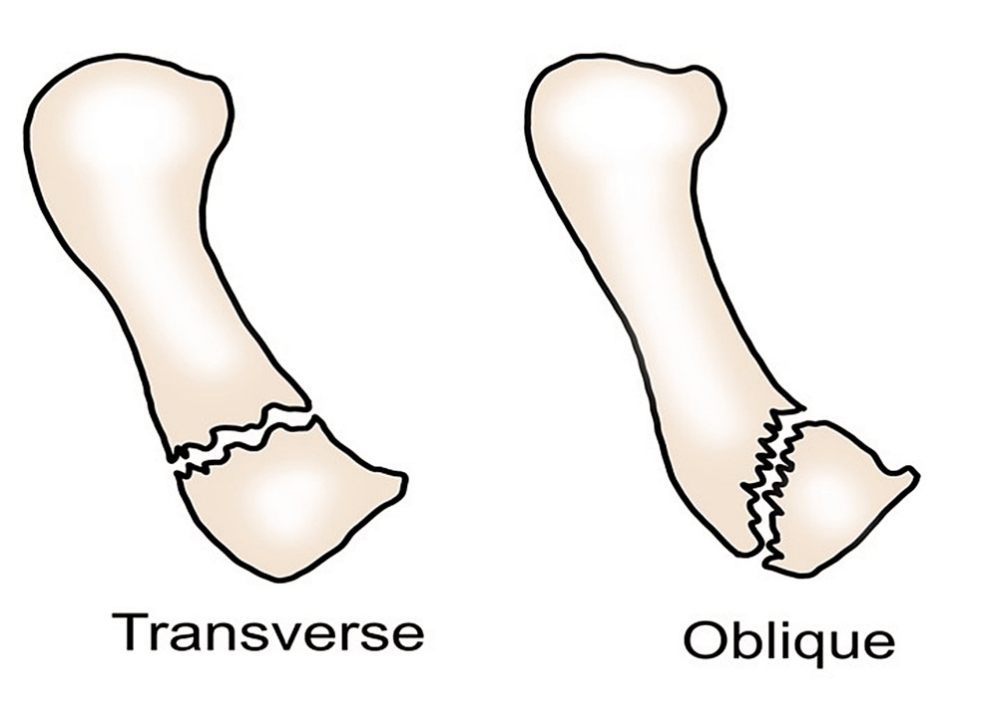
Transverse or oblique fracture extra-articular.

Extra-articular fracture can occur in two different parts, either in a short transverse or oblique direction (Figure 21). The inclined posterior ligament helps anchor the adjacent structures to the trapeziometacarpal joint. The movement of the adjacent APL, which is closely embedded, has an impact on these areas. The posterior ligament of the inclined bone provides stability and balance. However, the medial thenar muscle, which typically covers the first palmar plate, can act as a substitute and pull the distal part toward the proximal part. If left untreated without proper imaging, these fractures may decrease the initial plate space and result in a conflicting description of the opposite displacement of the distal part [65].

The size reduction of a specific fracture like an extra-articular fracture does not need to be precise. However, it is important to maintain the aperture of the first locking plate, even though opening the column of the thumb is typically done to achieve this reduction, there is no guarantee. Some authors suggest that extra-articular fractures with a motion angle of less than 30° can be managed and corrected externally using a splint [2]. However, most authors indicate that extra-articular fractures with a motion angle greater than 30° typically require surgery to avoid narrowing of the initial fiber line. Fixation of the trapeziometacarpal joint may seem to yield good results for some individuals [61], but the risk of joint damage resulting from iatrogenic causes appears disproportionate to the extra-articular fracture. Osteosynthesis is recommended by indirect percutaneous fixation between the bone with direct insertion into the ascending bone marrow or by using a small open plate. The prognosis for extra-articular fractures is generally better than intra-articular fractures [13], as the T-shaped locking plate does not prevent the occurrence of a second episode of the disease [66].

## Conclusions

Acute thumb injury can present with various symptoms. Therefore, it is essential to restore the opposing function of the thumb. Based on our experience, surgery is often necessary for the treatment of this condition, rather than conservative approaches. Its primary goal is to restore the mobility of the first carpometacarpal joint.
